# HEPA/Vaccine Plan for Indoor Anthrax Remediation

**DOI:** 10.3201/eid1101.040635

**Published:** 2005-01

**Authors:** Lawrence M. Wein, Yifan Liu, Terrance J. Leighton

**Affiliations:** * Stanford University, Stanford, California, USA;; † Children's Hospital Oakland Research Institute, Oakland, California, USA

**Keywords:** research, HEPA filter, anthrax, mathematical model, bioterrorism, remediation, vaccine

## Abstract

We developed a mathematical model to compare 2 indoor remediation strategies in the aftermath of an outdoor release of 1.5 kg of anthrax spores in lower Manhattan. The 2 strategies are the fumigation approach used after the 2001 postal anthrax attack and a HEPA/vaccine plan, which relies on HEPA vacuuming, HEPA air cleaners, and vaccination of reoccupants. The HEPA/vaccine approach leads to few anthrax cases among reoccupants if applied to all but the most heavily contaminated buildings, and recovery is much faster than under the decades-long fumigation plan. Only modest environmental sampling is needed. A surge capacity of 10,000 to 20,000 Hazmat workers is required to perform remediation within 6 to 12 months and to avoid permanent mass relocation. Because of the possibility of a campaign of terrorist attacks, serious consideration should be given to allowing or encouraging voluntary self-service cleaning of lightly contaminated rooms by age-appropriate, vaccinated, partially protected (through masks or hoods) reoccupants or owners.

## Abstract 2

A mathematical model suggests that a HEPA/vaccine approach is viable for most buildings after a large-scale anthrax attack.

In addition to killing 5 of its 11 victims, the 2001 anthrax attack on the U.S. Postal Service and federal facilities also contaminated a number of buildings. The U.S. government spent several hundred million dollars recovering buildings with large-area contamination by using chlorine dioxide fumigation. The last of these federal facilities, the Hamilton New Jersey Mail Sorting Facility, is not expected to reopen until early 2005, >3 years after the attack (*1*). A large-scale aerosol attack in a major metropolitan area could deny access to a portion of a city for years, with substantial economic and social consequences. While outdoor remediation would be challenging, the absence of sporicidal UV irradiation makes indoor remediation a particularly daunting task. Nonetheless, no federal agency has taken ownership of the wide-area remediation problem (*2*). A proactive plan to recover affected buildings quickly, safely, inexpensively, credibly, and with minimal collateral damage needs to be developed before such an event (*2*). To advance the analysis of these recovery options, we propose and evaluate a very simple HEPA/vaccine plan, where HEPA air cleaners continuously clean the indoor air and Hazmat workers use HEPA vacuums to clean the floors, walls, ceilings, and room contents on a twice-a-day basis; HEPA filters are 99.97% effective for 0.3-μm particles (*3*), which are 5–10 times smaller than a typical anthrax spore. In addition, residents are vaccinated before reoccupying the buildings. This strategy hypothesizes a nonzero standard for spore contamination and modest pre- and postremediation environmental sampling (in contrast, >5,000 negative environmental samples were taken after the fumigation of the Brentwood mail-processing facility [4]).The plan employs no sporicides, such as sodium hypochlorite (household bleach) or hydrogen peroxide, which can cause collateral damage to many hard surfaces, and does not discard carpets or furniture, which would generate profound solid waste problems. Using a hypothetical release in lower Manhattan, we compare the HEPA/vaccine and chlorine dioxide fumigation remedial options, in terms of anthrax cases among reoccupants, cost, and recovery time. No attempt is made to estimate the number of cases of cutaneous and gastrointestinal anthrax, which are less apt to be fatal. Although we focus on anthrax remediation, our framework may also be useful for indigenous agents of public health concern (e.g., tuberculosis, *Streptococcus*).

## Materials and Methods

**Figure 1:**
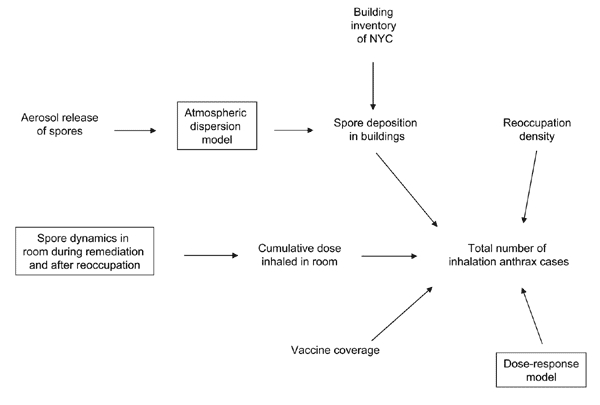
Graphic overview of the mathematical model. Mathematical submodels are in boxes. NYC, New York City.

**Figure 6:**
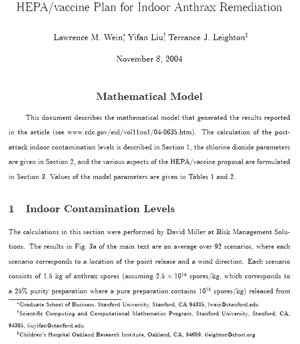
Model. This document describes the mathematical model that generated the reported results. The calculation of the postattack indoor contamination levels is described in Section 1, the chlorine dioxide parameters are given in Section 2, and the various aspects of the HEPA/vaccine proposal are formulated in Section 3. Values of the model parameters are given in Tables 1 and 2. Download PDF (245 KB, 26 pages).

A mathematical model (Figure 6) was used to evaluate the HEPA/vaccine (Figure 1) and fumigation modalities. In the model, 1.5 kg of anthrax spores is released outdoors in lower Manhattan from a height of 2 m. We considered 92 different scenarios in total, depending upon the release location and the wind direction. A building inventory of lower Manhattan (*5*) and an atmospheric dispersion model (*6*) were used to calculate the concentration of spores in each building in the exposed region. We assumed that postattack environmental sampling and plume analysis allow at least some of the "exposed region" to be correctly diagnosed within 1 week after the attack, at which time remediation begins. We also assumed that by day 7, outdoor contamination would have subsided to the point where it did not affect indoor spore concentrations.

Since chlorine dioxide fumigation eliminated all detectable spores from the Hart Senate Office Building and several mail-sorting facilities, we assumed that it successfully eliminates all spores in the buildings of our model. In the 2001 attack, chlorine dioxide was used to decontaminate the 700-km^2^ Brentwood postal facility, which took 1 year at a cost of $130 million (*4*); further discussion of this cost estimate appears in the mathematical model (Figure 6). Because the technology was new, we assumed that 50% of the cost was a 1-time investment in technology development. We further assumed a 90% learning curve in both cost and time (at this time, only a small number of companies possess chlorine dioxide expertise); i.e., each time the area of anthrax decontamination doubles, the marginal cost and time are reduced by 10%.

**Figure 2:**
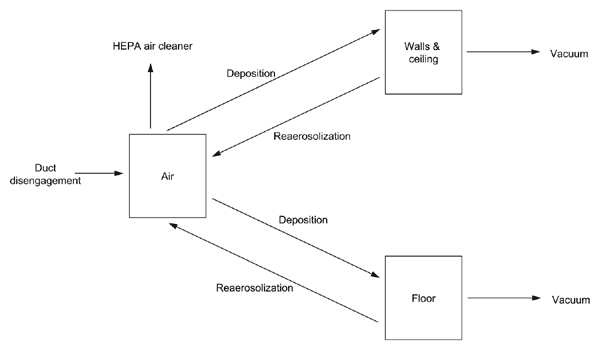
Graphic depiction of the compartments in the differential equation model and the spore movement among compartments.

To assess the HEPA/vaccine plan, we developed a differential equation model (Figure 2) of the spore dynamics within a generic 12x12x8-ft room in a building in the exposed region. The model measures the evolution of spore concentration in the air, on the room surfaces, and in the HVAC (heating, ventilation, and air-conditioning) system. A small fraction of spores adhere to the HVAC ducts as they enter the building, and then become slowly disengaged and enter the room. Rather than build multizone models of each building (*7*), we assumed that each room received air from a duct that is 50-m long, contains 360° of curvature, and has an air velocity of 1,000 ft/min. We implicitly assumed that all rooms within a building are remediated simultaneously, so as to minimize the effect of inter-room contamination within a building. Airborne spores in the room deposit on the room surfaces at a certain rate, and spores on the room surfaces, particularly the floor, reaerosolize at a rate that depends on the amount of activity in the room; more reaerosolization occurs during surface cleaning and reoccupation. The deposition rates and reaerosolization rates were derived by using data from the Hart Senate Office Building (*8*). HEPA air cleaners (achieving 10 air changes per h, possibly with the aid of dilution ventilation from the HVAC system) are used continually during the remedial period, which involves successive rounds of testing and vacuuming until *^n^*_5 _ postcleaning samples suggest that the floor spore concentration in the room is below the target level ^c̅^_ƒ_; this approach is reminiscent of that taken during the asbestos remediation after the World Trade Center collapse (*9*). Rather than use a spatial model to capture spatial heterogeneity of spores within a room, we simply assume that the floor samples are log normally distributed, where 95% of within-room samples at a fixed point in time are within 1 order-of-magnitude (i.e., within -1 / √1̅0̅  and √1̅0̅ of the median), which is consistent with the sample variability in the Hart Senate Office Building (*8*). That is, in the initial testing of *^n^*_5_ samples, we estimate the number of 2-h vacuumings of the room's surfaces and contents that are required to achieve the target concentration ^c̅^_ƒ_. After these vacuumings, a new set of *^n^*_5_ samples are taken. If the estimated concentration from these new samples is below ^c̅^_ƒ_ then remediation ceases; otherwise, another round of vacuuming and testing is performed. Consecutive vacuumings are 48 h apart, and testing (if needed) occurs midway between these 2 vacuumings, both to allow reaerosolized spores to resettle before testing and to permit the testing results to be received before the next scheduled vacuuming. We varied the 2 decision variables *^n^*_5_ and ^c̅^_ƒ _to explore the tradeoffs among our performance measures.

After the floor concentration is believed to have dropped below ^c̅^_ƒ _each generic room is reoccupied by 1 person for 12 h per day. After reoccupation, a portable HEPA air cleaner (at 3 air exchanges per h [10]) is used for 12 h every day, and 10 min of floor vacuuming occurs weekly at half the estimated efficiency of the remedial vacuuming. We assumed that 85% of reoccupants are successfully vaccinated and will not become infected, regardless of the spore concentration in the room. The remaining 15% represent infants, the elderly, the immunocompromised, and persons for whom vaccination is contraindicated, who are assumed to have a dose-response curve that correspond to the lowest 30% of the probit dose-response model (*11*) with a 50% infectious dose (ID_50_) of 8,000 spores (*12*) and a probit slope of 0.7 (*13*); e.g., the ID_15_from the probit model in (*11*) (i.e., 253 spores) would infect half of the unvaccinated population. Here, ID_50_ denotes the dose that infects half of the population; because inhalational anthrax is nearly always lethal (in the absence of treatment), the ID_50_ coincides with the 50% lethal dose (LD_50_). The differential equation model is used to measure the cumulative number of spores inhaled by each reoccupant in a 10-year period. Combining these cumulative doses, the dose-response model, the atmospheric dispersion model, and the population density of reoccupants allows us to compute the total number of inhalation anthrax cases.

The cost of the HEPA/vaccine plan includes $75/h for each Hazmat worker, who spends 4 h per 10-h shift vacuuming and the remaining 6 h resting, rehydrating, and handling protective gear; a $250 portable HEPA air cleaner for each 12x12x8-ft room; $25 for each environmental sample, which includes the costs for sampling, shipping, and laboratory testing; and $20 to vaccinate each person. If residents are vaccinated regardless of the remediation/reoccupation policy, the vaccination cost should be omitted from the comparison. The remediation time for the HEPA/vaccine plan was computed by assuming that 1,000 Hazmat workers (using level C protection) are available to perform remediation 10 h per day, which is ≈3 times larger than the labor force used at the Brentwood and Hart buildings, and that 200 samplers can each perform 24 samples in 4 h plus have 6 h for donning and removing protective gear, rest, and rehydration. The bottleneck for the total remediation time can be either sampling or vacuuming, depending upon the values of the concentration threshold (^c̅^_ƒ_) and the number of samples per round (*^n^*_5_).

## Results

**Figure 3:**
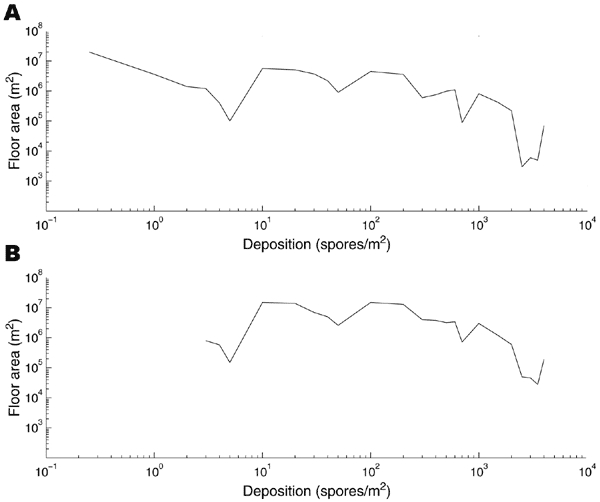
The amount of indoor floor area in lower Manhattan (vertical axes) that is contaminated at various anthrax concentration levels (horizontal axes) as a result of an outdoor release of 1.5 kg of anthrax spores. Plot A, an average of 92 scenarios (9 release locations in Manhattan times 8 wind directions, plus 20 release locations on the outskirts of Manhattan). Plot B, provides similar information for the scenario that generated the largest total area of contamination.

**Figure 4:**
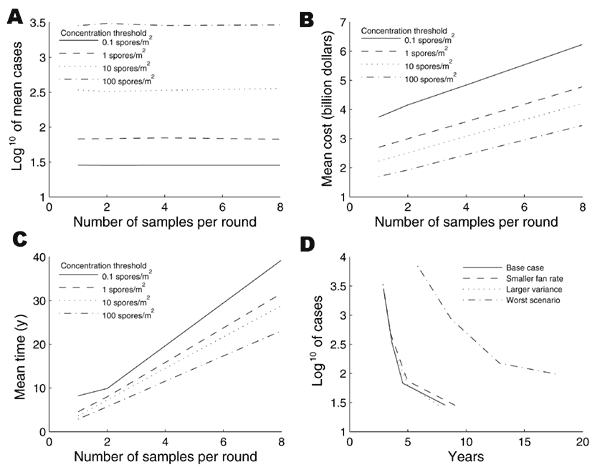
Performance of the HEPA/vaccine plan under the base-case scenario. The horizontal axes in plots A-C are the number of floor samples per round (). Each of plots A–C have 4 curves, 1 for each value of the floor concentration threshold (). Cleaning stops after the estimated floor concentration from samples per room is below the threshold . The vertical axes in plots A-C are A) the mean number of inhalation anthrax cases, B) the mean cost, and C) the mean recovery time. In plots A-C, the concentration threshold () has a much bigger impact than the number of samples per room () on these 3 performance measures. Plot D shows the tradeoff of anthrax cases versus recovery time in the base case. The number of samples per room is assumed to be = 1 in this plot, which is derived from plots A–C. Plot D also contains tradeoff curves for 3 sensitivity analyses: a lower air-cleaning rate, increased sampling variability of spore concentration, and the most severe of the 92 cases depicted in Figure 3B. This plot shows that the cases versus time tradeoff curve is very insensitive to changes in the air-cleaning rate and sampling variability.

We averaged the 92 scenarios to obtain a base case. Figure 3A shows the depositional distribution averaged for the 92 scenarios, i.e., the number of square meters of indoor floor area that are contaminated at various levels. The particular forms of dips and peaks in Figures 3A and 3B are due to the irregular spatial distribution of tall buildings relative to the release location that caused the most indoor contamination. The total contaminated area in this average scenario is 5.73 x 10^7^ m^2^, which is >4 million 12x12x8-ft rooms. For this base-case scenario, the fumigation plan costs $2.7 billion and takes 42 years. Figures 4A–C express the expected number of cases, cost, and time of the HEPA/vaccine plan for the base-case scenario in terms of the floor concentration threshold (^c̅^_ƒ_) and the number of floor samples per round (*^n^*_5_). Because of the random sample measurements, 50 simulations were performed to estimate each of the points in Figure 4, and the 95% half-confidence intervals are < 0.05 times the sample mean in all cases. Figure 4A shows that the mean number of anthrax cases is nearly independent of the number of samples per round, and drops from ≈3,000 cases when the floor concentration threshold is 100 spores/m^2^ to 28 cases when the floor concentration threshold is 0.1 spores/m^2^. To put these numbers in perspective, we also found that 15,760 cases would occur if no cleaning was performed (i.e., ^c̅^_ƒ _^=∞^). The total cost in Figure 4B varies from $1.7 billion to $6 billion and depends more on the spore concentration threshold than the number of samples per round. The mean remediation time ranges from 2.9 years to 39.3 years; since there are approximately 4 million rooms and vacuuming can be done at the total rate of 2,000 rooms/day, it would take 5.5 years to clean each room once. Vacuuming dictates the total remediation time in Figure 4C when *^n^*_5_= 1 and ^c̅^_ƒ_ = 0.1 or 1, and sampling is the bottleneck for the other values of *^n^*_5_ tested. Because using *^n^*_5_> 1 increases the cost and time without decreasing anthrax cases, we focus in Figure 4D on the cost versus time tradeoff by fixing *^n^*_5_=1.

**Figure 5:**
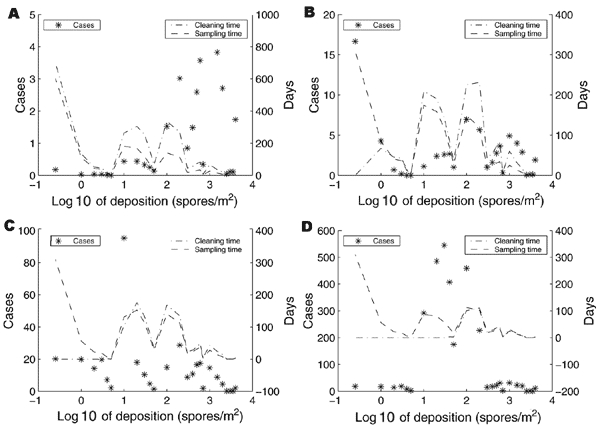
The horizontal axes in these 4 plots give the original room deposition level before remediation begins. These plots show how the total number of anthrax cases (the stars and the left vertical axes) are distributed across room deposition levels, e.g., in plot A, most of the anthrax cases occur in rooms with original deposition levels >100 spores/m^2^. Similarly, the 2 curves and the right vertical axis of each plot show how much time is spent cleaning and sampling in rooms of various deposition levels. These 4 plots are identical except that the spore concentration threshold in spores/m^2^ (^c̅^_ƒ_), which dictates when remediation is stopped, is A) ^c̅^_ƒ _= 0.1; B) ^c̅^_ƒ _ = 1; C) ^c̅^_ƒ _ = 10; D) ^c̅^_ƒ _ = 100. These plots motivate the hybrid policy, which fumigates heavily contaminated rooms and uses the HEPA/vaccine approach in lightly contaminated rooms.

Figure 5 depicts the mean cases and mean remediation time according to the amount of original spore deposition in the rooms. Figure 5A suggests a hybrid strategy that fumigates heavily contaminated rooms (>100 spores/m^2^) and uses the HEPA/vaccine approach for lightly contaminated rooms (<100 spores/m^2^). This hybrid approach results (on average) in only 2 anthrax cases, and the mean remediation time for the lightly contaminated rooms is 5.9 years. It takes 8.4 years to fumigate the highly contaminated rooms. Hence, the total remediation time ranges from 8.4 to 14.3 years, depending upon whether different workers are involved in the 2 decontamination modalities. For the 3 other threshold levels pictured in Figures 5B–5D, many of the anthrax cases occur right at the cutoff point, which is due to the tail behavior of the spore depositional distribution in Figure 3A. The hybrid strategy is not as helpful with these higher threshold levels; e.g., using a threshold of 1 spore/m^2^ to decide between fumigation and vacuuming in Figure 5A, the plan would vacuum for 2 years and fumigate for 28 years.

### Sensitivity Analyses

A number of aspects of the model contain considerable uncertainty: the cost and time of the fumigation plan, the indoor spatial deposition after an attack, the reaerosolization and deposition rates inside a room, spore dynamics in a duct, air-cleaning efficacy, vacuum efficacy, Hazmat logistics, the spatial heterogeneity in sampling, vaccine coverage, and the low end of the dose-response curve. Before discussing each of these 10 variables in turn, we note that our general approach to these uncertainties is to be conservative with respect to assessing the HEPA/vaccine option; i.e., we err on the side of overstating the mean number of anthrax cases that would result under this approach or understating the cost and time of the fumigation plan.

Although fumigation was successful during the cleanup after the 2001 postal attack, the fumigation of a skyscraper is a challenge that has yet to be tackled. Given the 42 years it would take to fumigate the exposed area, an alternative technology could be developed.

The estimated indoor spatial deposition contains orders-of-magnitude of uncertainty, depending upon the size of the release, the spore characteristics (e.g., dry versus wet, size, purity, viability, surface electrostatic properties), the weather conditions, building and canopy terrain in lower Manhattan, building HVAC infrastructure, and whether or not windows and vents were open. The goal of the atmospheric modeling is neither to accurately predict the probability distribution of indoor spatial concentrations for a possible future attack (such an attempt would be greatly limited by the irreducible uncertainty in the release size) nor to provide postattack situational awareness (which would require a much more detailed spatial model), but rather to generate a comparative set of plausible scenarios to evaluate remediation strategies before an attack. Hence, we focused on the average of 92 plausible scenarios. To give some sense of the upper range, we present in Figure 4D the results from the most severe of the 92 scenarios; the deposition distribution from this scenario appears in Figure 3B. This scenario contaminates ≈7 million rooms and requires 17.7 years to reduce the number of cases to 98.

Because air and surfaces are concomitantly remediated, the number of anthrax cases is rather insensitive to the reaerolization and deposition rates in the room.

The large uncertainty with respect to duct modeling led us to adopt a worst-case approach and use the spore disengagement rate that maximizes the number of anthrax cases. Many new buildings and some retrofitted older buildings have HEPA filters built into the HVAC system (*14*), which would largely eliminate the risk for spore disengagement.

We have focused on portable air cleaners, whereas dilution ventilation, in which 15%–25% of the total airflow rate consists of outside airflow (*15*), may also play a key role in remediation. Figure 4D also presents results when we reduce the air-cleaning rate during remediation from 10/h to 3/h. The latter quantity, which can be achieved with an off-the-shelf air cleaner and an open window (*10*), generates only a minor change in the cases versus time tradeoff curve.

To the extent that reaerosolized spores resettled before or during postvacuum testing in the referenced study (*16*), we may have underestimated the vacuum efficacy. We conservatively assumed that all floors are carpeted and that sporicides such as sodium hypochlorite, hydrogen peroxide, or foams (*17**,**18*), which are much more effective than vacuuming for hard surfaces, are not deployed.

Our assumption that each Hazmat worker has 4 productive hours of work per day underestimates the rate that could be achieved over a several-week time frame but is prudent over a longer period of time and would help avoid worker fatigue and burnout.

Because the amount of spatial heterogeneity of spores in a room is difficult to assess, we considered the case where 95% of samples within a room fall within 2 orders of magnitude rather than 1. Figure 4D shows that the effect of this increased sampling variability is negligible and that the optimal amount of sampling did not change relative to the base case.

As noted in section 3.8 of the mathematical model, our 85% vaccine coverage of reoccupants may be a considerable underestimate. No age groups are being left behind in the plans for the next-generation anthrax vaccine, and persons with weak immune systems may achieve partial protection.

We considered a cumulative dose during a 10-year period, whereas infection may be a result of a challenge over a shorter time horizon; our overestimate of cases is very modest because of the exponential decreases in spore concentration during the reoccupation period, and changing the horizon from 10 years to 6 months led to a negligible (<1%) reduction in cases. Our dose-response model assumed that the 15% unvaccinated population comes from the most vulnerable 30% of a widely used probit model, which itself has been criticized for greatly overestimating the number of cases at the lower end of the curve (*19*). If we used 95% vaccine coverage with the remaining 5% sampled from the lower 50% of the probit model, then the number of anthrax cases with ^c̅^_ƒ _ = 10 spores/m^2^ and *^n^*_5_ = 1 sample per round would be reduced from 341 to 72. Even within the class of probit models, others have used a probit slope twice as steep, which results in many fewer cases (*20*). If we use a probit slope of 1.4 rather than 0.7, then the mean number of cases with ^c̅^_ƒ _= 10 spores/m^2^ and *^n^*_5_ =1 sample per round decreases from 341 to 3 x 10^-5^, which highlights the value of further research into the low end of the dose-response relationship. However, in the mathematical model we note that the slope of 0.7 is more consistent with data from the 2001 anthrax attack. Dahlgren et al. (*21*) estimated that goat-hair mill workers routinely inhaled about 500 (<5 μm) anthrax spores per shift without accompanying illness or death, raising the possibility (although no subsequent work on this topic has been published) that chronic low-level exposure might induce adaptive or innate immunity. In any case, adaptive or innate immunity is unlikely to occur in the 15% of people in our model who are not successfully vaccinated. One assumption that is not conservative is that people reoccupy these rooms for 12 h per day. A small fraction of people may work at home, stay at home most of the day, or work and live in different buildings within the exposed region. We are underestimating the inhaled doses for these people by a factor of 2. Nonetheless, taken together, the numerical results reported here may overstate the actual number of anthrax cases by at least 1 order of magnitude, and perhaps many.

## Discussion

The base-case release, which is an average of 92 different scenarios under various weather conditions and locations in lower Manhattan, contaminates the equivalent of 4 million 12x12x8-ft rooms. Our analysis suggests that an outdoor release would generate a more diffuse depositional distribution of spores than an indoor attack: we estimate that ≈10,000 spores/m^2^ were deposited in parts of the Hart Senate Office Building (section 3.2 of the mathematical model), which is considerably higher than the concentrations in Figure 3. As an alternative to a multidecade fumigation effort, the HEPA/vaccine plan appears capable of substantially reducing the number of anthrax cases but would require ≈8 years with the current estimated Hazmat labor pool. Both plans would require several billion dollars in direct costs. The HEPA/vaccine plan eventually experiences diminishing returns: from a base of 341 expected cases after 3.6 years of remediation, another year is required to reduce the mean number of cases to 67, but then an additional 3.6 years and $1 billion are needed to reduce the mean number of cases to 28. A hybrid HEPA/vaccine/fumigation plan, in which lightly contaminated buildings receive the HEPA/vaccine approach and heavily contaminated buildings are fumigated, could eliminate almost all of the anthrax cases. The required remediation time would be 8.4–14.3 years, depending upon whether the same Hazmat personnel carried out both operations.

A key finding of our study is that only a moderate amount of sampling appears to be required. In theory, additional sampling reduces type I and type II errors, thereby avoiding anthrax cases in rooms that were inadvertently thought to be sufficiently safe, and reducing unnecessary remediation of rooms that were mistakenly perceived as overly contaminated. However, the number of anthrax cases was essentially independent of the number of room samples per round, as long as at least 1 sample was taken. Indeed, with current vacuuming and sampling capacity, the only impact from taking >1 sample per 12x12x8-ft room is prolonged remediation and increased cost. However, in the absence of exhaustive environmental testing, on-site coordinators need to validate that work is performed according to the required standards (i.e., vacuuming is actually being done for the specified number of minutes/m^2^).

Our results have several implications. First and foremost, field tests with simulants are required to accurately assess the real-world spore reduction that can be achieved—and the number of vacuumings required—by this HEPA/vaccine approach. If field tests confirm the model predictions, then the concentration threshold ^c̅^_ƒ_, the number of samples per round *^n^*_5_, and the level of concentration that requires fumigation versus vacuuming should be determined with greater precision. These threshold values should be chosen so that the reoccupant risk level (in terms of quality-adjusted life years) is consistent with those for other hazards (e.g., asbestos, radiation).

Large-area urban remediation strategies must confront a number of difficult issues, the most important of which is surge Hazmat capacity. We have assumed that remediation and vaccination are initiated simultaneously 1 week after the attack. The initial vaccination of reoccupants would require ≈1 week; protective immunity is believed to develop at 35 days after initial vaccination (*22*). Hence, residents will be able to reoccupy buildings by 42 days after remediation is initiated. Presumably, most reoccupants would receive prophylactic antimicrobial agents because they would have been in these building during or soon after their exposure. Consequently, some of these residents may be interested in moving back in even earlier. Considering that 8.2 years is required to carry out the HEPA/vaccine plan in the base-case scenario, this reoccupancy delay may be viewed by the major stakeholders as unacceptable. Our analysis assumes the availability of 1,000 Hazmat personnel, compared to the 300 Hazmat workers (after attrition) used to perform the Brentwood cleanup and the roughly 3,000 licensed asbestos workers in New York State. To reduce the recovery delay from 8.2 years to 5 months requires a 20-fold increase in Hazmat labor, i.e., 20,000 personnel. To reduce the delay another 4-fold so as to allow reoccupation within 42 days is probably not realistic for this large-area scenario. Nonetheless, U.S. government coordination with the Hazmat, fumigation, and building protection industries—not just locally, but nationwide and perhaps including the U.S. military and key allies—would be necessary to guarantee available capacity and resources. In addition, scheduling theory (*23*) implies that aggregate waiting time for reoccupants can be minimized by remediating the least-contaminated buildings first (i.e., use the shortest expected processing time priority rule).

There are other aspects to optimizing surge remediation and recovery capacity. Just as the worried well caused a surge in ciproflaxin sales in 2001, many people outside of the exposed region will attempt to buy HEPA air cleaners and vacuums. Hence, demand will come not only from the exposed area but also from surrounding regions. In the same way that the U.S. government is working with pharmaceutical companies to provide surge capacity of medical countermeasures (including anthrax vaccine) in the event of a biologic attack, it needs to develop cooperative agreements with building protection service companies so that equipment shortages do not block the critical path to recovering the exposed area.

Another key aspect of a detailed plan is exception management: the HEPA/vaccine plan will not work for 100% of the buildings in the exposed area. More aggressive remediation of critical assets (hospitals; nursing homes; daycare centers; emergency response facilities; electrical, water and sanitation facilities; transportation facilities) will be desirable. Some nonresidential buildings (such as the buildings contaminated in the 2001 attack) have extremely high ceilings, and achieving a high air-exchange rate in these spaces may be not be feasible with portable air cleaners. Another confounding issue is visitors to the impacted region. In the aftermath of a catastrophic anthrax attack, the public would expect nationwide voluntary mass vaccination. Visitors to the exposed areas should be offered an anthrax vaccine, and guidelines for unvaccinated visitors should be developed. Also, because the spore concentration continues to decrease exponentially during reoccupation (but not during semiquiescent periods), more vulnerable residents might delay their reoccupation until several months after the other residents. A significant logistical issue is the disposal of contaminated carpets, furniture, and other household goods. Some reoccupants will insist on discarding these items, even after they have been heavily cleaned. Reoccupant education and outreach measures, including perhaps temporal or financial disincentives for disposal, need to be taken to avoid overwhelming solid waste disposal capacity. Emergency plans (e.g., medical incinerator capacity) should be developed for the HEPA vacuum bags and other items that need to be discarded during remediation. Another difficult issue is postevent building maintenance, particularly of HVAC systems, which must minimize spore reaerosolization during maintenance and disposal of old ducts. Safe procedures to rid ducts of asbestos (asbestos fibers are roughly the same size as anthrax spores, but the U.S. Environmental Protection Agency limit for asbestos is 900 fibers/m^3^ [9], which is larger than the postremediation spore concentrations considered here) and other materials have been developed (*24*); the important point is that HVAC cleaning should not block the critical path to reoccupation but rather should be performed asynchronously in a low-intensity manner over many years.

In summary, this study suggests that a HEPA/vaccine approach is viable for most buildings after a large-scale anthrax attack. This outcome is dependent on a qualitative increase in surge Hazmat remediation capacity to reduce the recovery delay to a level that would not invite permanent mass relocation. Detailed mass remediation plans need to be developed now; as noted by Danzig (*2*), without such a plan we are inviting economic and social disruption. Ultimately, the extent of restoration and sampling will be dictated by the reoccupants and building owners, and hence risk communication will be of the utmost importance. Inconvenience and cost may force relaxation of standards, and some thought should be given to whether voluntary "self-service" cleaning of minimally contaminated rooms by age-appropriate, vaccinated, partially protected (e.g., with N95 masks) reoccupants or owners would be allowed or encouraged. Indeed, in the face of a campaign of terrorist attacks (*2*), this self-service approach, with more effective masks or hoods, may be the only feasible response. Finally, a safe, effective, single-dose vaccine would have a profound impact on mitigating the undesirable consequences of this scenario.

## Supplementary Material

Figure 6 Figure 6 in PDF format
