# Supplementary material for: HEPA/Vaccine Plan for Indoor Anthrax Remediation
Source: Emerg Infect Dis. 2005 Jan;11(1):69–76. doi: 10.3201/eid1101.040635 (PMC3294362; doi:10.3201/eid1101.040635)
Supplement: Figure 6 — in PDF format [file 04-0635-F6.pdf]

HEPA/vaccine Plan for Indoor Anthrax Remediation

Lawrence M. Wein,[*] Yifan Liu,[†] Terrance J. Leighton[‡]

November 8, 2004

Mathematical Model

This document describes the mathematical model that generated the results reported in the article (see www.cdc.gov/eid/vol11no1/04-0635.htm). The calculation of the post-attack indoor contamination levels is described in Section 1, the chlorine dioxide parameters are given in Section 2, and the various aspects of the HEPA/vaccine proposal are formulated in Section 3. Values of the model parameters are given in Tables 1 and 2.

1 Indoor Contamination Levels

The calculations in this section were performed by David Miller at Risk Management Solutions. The results in Fig. 3a of the main text are an average over 92 scenarios, where each scenario corresponds to a location of the point release and a wind direction. Each scenario consists of 1.5 kg of anthrax spores (assuming 2.5×10^{14} spores/kg, which corresponds to a 25% purity preparation where a pure preparation contains 10^{15} spores/kg) released from

[*]Graduate School of Business, Stanford University, Stanford, CA 94305, lwein@stanford.edu

[†]Scientific Computing and Computational Mathematics Program, Stanford University, Stanford, CA, 94305, liuyifan@stanford.edu

[‡]Children's Hospital Oakland Research Institute, Oakland, CA, 94609, tleighton@chori.org

a height of 2 m. For each of nine release locations in lower Manhattan, eight different wind directions were simulated. In addition, we included 20 other scenarios corresponding to releases on the outskirts of Manhattan, for a total of 92 scenarios. For each scenario, the SCIPUFF atmospheric dispersion model [1], which uses a Gaussian puff model with a boundary layer using default values in [1], a wind speed of 2 m/s and a decay rate of 1/s in the daytime and 0.1/s at night, computed the outside deposition in spores/m^2.

The building inventories in this analysis consist of all structures south of Central Park in New York City, using a database that contains accurate location, plan dimensions and number of floors for all buildings in this locale [2]. Indoor deposition levels were calculated by assuming that only a fraction of spores enters a structure. Because taller buildings are better sealed, we assume that the fraction of spores that entered the building and deposited in the rooms decrease with the number of floors in the building, and range between 0.05 and 0.4. We further assume that once inside a structure, spores are evenly distributed across the rooms of the structure. While this is an oversimplification, it appears that anthrax spores have the potential to quickly disperse throughout a large building [3]. As explained in §3.5, we also assume an additional small fraction of spores are deposited in the ducts.

We let $n(D)$ be the total number of square meters of indoor floor space that has a deposition of D spores/m^2 (see Figure 1a of the main text). We also define $A = \int_0^\infty n(D)\, dD = 5.73 \times 10^7$ to be the total number of square meters of contaminated indoor floor space.

2 Chlorine Dioxide Fumigation

Since chlorine dioxide fumigation eliminated all spores from the Hart Senate Office Building and several mail sorting facilities, we assume that it successfully eliminates all spores in these 92 scenarios. In the 2001 attack, chlorine dioxide was used to decontaminate the 700k m^2 Brentwood postal facility, which took one year at the estimated cost of \$130M [4]. The

final cost, including indirect costs, may be considerably larger than this estimate, and the USPS claims that the future cost of such an endeavor would be \$10-15M [5], even though earlier remediation estimates from the 2001 attack were far too optimistic [5, 6]. Because the technology was new, we assume that 50% of the cost was a one-time investment in technology development. We further assume a 90% learning curve in both cost and time (at this point in time, there are only two companies that possess the chlorine dioxide technology); i.e., each time the amount of square area of anthrax decontamination doubles, the marginal cost goes down by 10%. Hence, the total cost to fumigate A m^2 is $c_c \int_0^A x^{-0.152} dx$, where $0.152 = \ln 0.9/\ln 2$ and c_c is the cost to fumigate the first square meter. Solving \$65M $= c_c \int_0^{700{,}000} x^{-0.152} dx$ yields $c_c = \$609$. Similarly, the time required to fumigate A m^2 is $\tau_c \int_0^A x^{-0.152} dx$, where $\tau_c = 0.082$ hr is the time to fumigate the first square meter, and satisfies $\tau_c \int_0^{700{,}000} x^{-0.152} dx = 1$ yr. Substituting the parameters A, c_c and τ_c into these integrals reveals that the total fumigation cost is \$2.7B and the total fumigation time is 41.9 years.

3 HEPA/vaccine Approach

This section describes the modeling elements of the HEPA/vaccine approach. A dynamic compartmental model is formulated in §3.1, the surface deposition and reaerosolization parameters are derived in §3.2, the cleaning of surfaces and the air are described in §3.3 and §3.4, a duct analysis is performed in §3.5, the sampling and cleaning strategies are prescribed in §3.6, the post-reoccupation cleaning and cumulative dose are described in §3.7, vaccine coverage, efficacy and cost are stated in §3.8, the dose-response model is specified in §3.9, and the computation of cases, costs and total time is described in §3.10.

3.1 Dynamic Compartmental Model

We consider a well-mixed three-compartment model to assess the spore dynamics in a generic room of size $12 \times 12 \times 8$ ft, consisting of the spore concentrations in the air ($c_a(t)$ spores/m^3), on the walls and ceiling ($c_w(t)$, spores/m^2) and on the floor ($c_f(t)$ spores/m^2) at time t. The aggregation of the walls and ceiling into a single compartment is justified in §3.2. We denote the room volume by $V = 32.62$ m^3, the floor surface area by $A_f = 1.2(13.38) = 16.05$ m^2 and the surface area of the walls and ceiling by $A_w = 1.2(49.05) = 58.86$ m^2, where the 20% inflation factor of the surface areas accounts for the furniture and other contents in the room. The model captures the inflow of spores from contaminated ducts at rate $d(t)$, the adsorption of spores to the room surfaces at rate l_a, where a fraction $f_w(t)$ adheres to the walls and ceiling and the remaining fraction is deposited on the floor, and the reaerosilization from the surfaces at rate $r_f(t)$ from the floor and $r_w(t)$ from the walls and ceiling. The deposition fraction $f_w(t)$ and the reaerosolization rates are expressed as functions of time because they will vary depending on the activity conditions in the room, as explained in §3.2. Cleaning occurs via two first-order mechanisms: a portable HEPA filter with a fan reduces the airborne spore concentration at rate $k_a(t)$, and HEPA vacuuming of the ceiling, walls and floor (and, implicitly, all room contents, although there will be areas – e.g., individual pages of books – that are difficult to access by Hazmat workers) decreases the spore concentration of the walls and floor at rates $k_w(t)$ and $k_f(t)$, respectively. The cleaning parameters are function of time because some cleaning also occurs after reoccupation. We assume that all of the outdoor spores have been inactivated by the time that indoor remediation of the generic room begins, which is $\tau_d = 7$ days after the attack and taken to be time $t = 0$. Remediation lasts for T hours, during which time the fan and HEPA filter are in continuous use. For $t \in [0, T]$, let the indicator function $I_w(t) = 1$ if the wall is being vacuumed at time t and $I_w(t) = 0$ otherwise, and define $I_f(t)$ for the floor in an analogous fashion. The system dynamics are

given by

$$\underbrace{\dot{c}_a(t)}_{\text{air}} = \underbrace{d(t)}_{\text{duct}} + \underbrace{\frac{A_w r_w(t)}{V} c_w(t)}_{\text{reaerosolization}} + \underbrace{\frac{A_f r_f(t)}{V} c_f(t)}_{\text{reaerosolization}} - \underbrace{l_a c_a(t)}_{\text{deposition}} - \underbrace{k_a(t) c_a(t)}_{\text{cleaning}}, \tag{1}$$

$$\underbrace{\dot{c}_w(t)}_{\text{walls}} = \underbrace{\frac{V f_w(t) l_a}{A_w} c_a(t)}_{\text{deposition}} - \underbrace{r_w(t) c_w(t)}_{\text{reaerosolization}} - \underbrace{k_w(t) I_w(t) c_w(t)}_{\text{cleaning}}, \tag{2}$$

$$\underbrace{\dot{c}_f(t)}_{\text{floor}} = \underbrace{\frac{V(1 - f_w(t)) l_a}{A_f} c_a(t)}_{\text{deposition}} - \underbrace{r_f(t) c_f(t)}_{\text{reaerosolization}} - \underbrace{k_f(t) I_f(t) c_f(t)}_{\text{cleaning}}. \tag{3}$$

We determine the values of the parameters in (1)-(3), including the initial system state, in the next five subsections.

One dynamic aspect we fail to capture in (1)-(3) is that all rooms are assumed to start cleaning seven days after the attack, whereas some rooms will be cleaned later than that if there are not enough Hazmat laborers to clean all buildings simultaneously (see §3.10). However, the only term in our model that depends on the exact starting time of cleaning is the duct source term $d(t)$ in (1), and the relative magnitude of this term is too small (see §3.5) to have this simplifying assumption affect our qualitative conclusions.

3.2 Surface Deposition and Reaerosolization

In this subsection, we estimate the initial system state, the deposition parameters l_a and $f_w(t)$, and the reaerosolization parameters $r_w(t)$ and $r_f(t)$. We use data from Tables 2 and 4 in Weis *et al.* [7], who measured air and floor concentrations in the Hart Senate Office Building during simulated semi-quiescent and active conditions. During active conditions, they found 2800 spores/m^2 deposited on the floor and other horizontal surfaces, 11,000 spores/m^3 in the air near the floor, 707 spores/m^3 in the air near the breathing zone, and 75 spores/m^2 on the office dividers (i.e., walls). During semi-quiescent conditions, they

measured 171 spores/m^3 in the air near the floor. They also found very little change in vertical surface concentrations as a result of increased activity.

We first use these data to estimate the initial conditions, assuming that active conditions prevailed as the spores deposited during the hours after the silent attack. The ratio of wall-to-floor concentration is 75/2800=0.026. To estimate the ceiling concentration, we ignore the walls and use a simple one-dimensional reaerosolization model, where during active conditions a fraction $1 - f_a^a$ of the spores in the room stay on the floor, and the remaining fraction of spores are distributed in the air at height h according to the exponential function ae^{-ah}, and stick to the ceiling (at height $H = 8$ ft) according to $\int_H^\infty ae^{-ah}\,dh = e^{-aH}$. Using the data in [7], we solve $D(1 - f_a^a) = 2800$, $Df_a^a ae^{-0.1a} = 11,000$, and $Df_a^a ae^{-1.6a} = 707$, and get $D = 10,018.8$ spores/m^2 (the deposition in [7]), $f_a^a = 0.72$ and $a = 1.83$/m. Hence, the ceiling-to-floor concentration is $\frac{f_a^a e^{-aH}}{1-f_a^a} = 0.030$. Since the ceiling and wall depositions are very similar, we aggregate the ceiling and walls into a single compartment in (1)-(3). Using the average of 0.030 and 0.026, we derive the conditions soon after the attack to be $c_a(t) = 0$, $c_w(t) = 0.027D$, and $c_f(t) = 0.973D$, where D is the total deposition (spores/m^2) computed in §1. As explained in §3.5, the floor and wall concentrations when cleaning begins (at time 0) will include not only $0.027D$ and $0.973D$, respectively, but also some spores that originally adhered to the duct but disengaged from the duct and deposited on the walls and floor before time 0.

The surface absorption parameter l_a is equal to the surface area of the room, $A_s = 1.2[2(12^2) + 4(8)(12)]$ ft^2 = 806.4 ft^2 = 74.96 m^2, times the adsorption coefficient (in m/s), divided by the room volume, V. For particles of diameter larger than 2 or 3 μm – we assume the spores are $D_p = 3$ μm in diameter – the adsorption is dominated by gravimetric settling and the adsorption coefficient is taken to be the gravimetric settling velocity [8], which is

$$v_y = \frac{Cg(\rho_p - \rho)D_p^2}{18v_d}. \tag{4}$$

In (4), $C = 1.05$ is the Cunningham slip factor, $g = 9.81$ m/s^2 is the acceleration of gravity, $\rho = 1.184$ kg/m^3 is the density of air, $v_d = 1.83 \times 10^{-5}$ kg m^{-1} s^{-1} is the dynamic velocity under standard temperature and pressure, and $\rho_p = 283$ kg/m^3 is the density of anthrax spores, assuming 2.5×10^{14} spores/kg. These substitutions lead to $v_y = 7.93 \times 10^{-5}$ m/s. Taking the room surface area to be $A_s = 74.96$ m^2 and the room volume to be $V = 32.62$ m^3, we find that the surface absorption parameter is $l_a = 1.82 \times 10^{-4}$/s, which is in close agreement with experiments [9, 10].

The remaining three parameters, $f_w(t)$, $r_w(t)$ and $r_f(t)$, can take on one of two values, depending on whether active or semi-quiescent conditions prevail at time t. In particular, we assume conditions are active during surface cleaning, which is consistent with the observation that vacuuming may increase the rate of reaeorsolization [11], and are semi-quiescent throughout the remainder of the remediation period:

$$f_w(t) = \begin{cases} f_w^a & \text{if } I_w(t) = 1, \quad t \in [0, T); \\ f_w^s & \text{if } I_w(t) = 0, \quad t \in [0, T); \end{cases} \tag{5}$$

$$r_w(t) = \begin{cases} r_w^a & \text{if } I_w(t) = 1, \quad t \in [0, T); \\ r_w^s & \text{if } I_w(t) = 0, \quad t \in [0, T); \end{cases} \tag{6}$$

$$r_f(t) = \begin{cases} r_f^a & \text{if } I_f(t) = 1, \quad t \in [0, T); \\ r_f^s & \text{if } I_f(t) = 0, \quad t \in [0, T). \end{cases} \tag{7}$$

We simultaneously solve for these parameter values by assuming that the data in Weis *et al.* [7] represent an equilibrium state in either active or semi-quiescent conditions. That is, we set the left sides of (1)-(3) to 0, ignore the duct term and the cleaning terms in these equations, set $c_a(t)$, $c_w(t)$ and $c_f(t)$ to their equilibrium values, and then solve for $f_w(t)$, $r_w(t)$ and $r_f(t)$. We let $c_a(t)$ be the average air concentration, which is $\frac{Df_a^a}{H}\int_0^H ae^{-ah}\,dh = Df_a^a(1 - e^{-aH})/H$, and as before let $c_w(t) = Df_a^a e^{-aH}$ and $c_f(t) = D(1 - f_a^a)$. Substituting these expressions into (1)-(3) gives (the deposition level D cancels out)

$$0 = \frac{A_w f_a^a e^{-aH}}{V} r_w(t) + \frac{A_f(1 - f_a^a)}{V} r_f(t) - \frac{l_a f_a^a(1 - e^{-aH})}{H}, \tag{8}$$

$$0 = \frac{Vl_a f_a^a (1 - e^{-aH})}{A_w H} f_w(t) - f_a^a e^{-aH} r_w(t), \tag{9}$$

$$0 = \frac{Vl_a f_a^a (1 - e^{-aH})}{A_f H} (1 - f_w(t)) - (1 - f_a^a) r_f(t). \tag{10}$$

We define the three parameter values by (f_w^a, r_w^a, r_f^a) under active conditions and by (f_w^s, r_w^s, r_f^s) under semi-quiescent conditions. Under active conditions, we substitute the values derived earlier in this subsection, $f_a^a = 0.72$ and $a = 1.83$/m, into (8)-(10). Under semi-quiescent conditions, we maintain $a = 1.83$/m but use $f_a^s = 0.02$, which solves $Df_a^s e^{-0.1a} = 171$ with $D = 10,018.8$ spores. However, the systems of equations (8)-(10) is singular and has rank two, and hence we need another independent equation to solve for the three parameter values. Under active conditions, we impose the extra condition

$$r_w^a = r_f^a \tag{11}$$

because all the surfaces are being vacuumed. Under semi-quiescent conditions, we add the equation

$$r_w^s = r_f^s \tag{12}$$

because none of the surfaces experience much activity. The three parameter values that solve (8)-(11) and (8)-(10), (12), respectively, are given in Table 1.

3.3 Surface Cleaning

Sodium hypochloride (household bleach), diluted with water to reduce the pH from 12 to 7, can achieve a 4-log decrease of *Bacillus* spores in 30 minutes [12], which gives a first-order killing rate of 0.307/min. Hydrogen peroxide (25.8%), which should result in less mucosal irritation than sodium hypochloride, can achieve a 5-log reduction in 15 minutes at room temperature (first-order killing rate is 0.768/min) [13]. The sporicidal efficiency of both agents may be reduced by the presence of organic matter [14]. Newer sporicidal foams

[15, 16] and emulsion surfactants [17] also appear to be effective, and may cause less damage to the environment and/or the treated surfaces than the two traditional agents.

We have chosen to use a simpler, if less effective, surface cleaner – a HEPA vacuum – because sodium hypochloride and hydrogen peroxide may cause undesirable collateral damage to room contents and sporicidal foams are difficult to remove from hard surfaces. Unfortunately, there is no data on the efficiency of HEPA vacuuming for anthrax spores. Because anthrax spores are roughly the same size as asbestos fibers, we use asbestos data to estimate the vacuuming efficiency. HEPA-filtered hot water extraction achieved a 69% reduction of asbestos fibers in carpets after vacuuming 46.5 m^2 for 65 min [18]. We assume that walls and ceilings would achieve about a 90% reduction for the same amount of vacuuming. A 90% spore reduction on the floor of our generic room requires $\frac{-\ln 0.1}{-\ln 0.31}\frac{65\text{min}}{46.5\text{m}^2}A_f = 44.2$ min, and a 90% reduction on the walls and ceiling requires $\frac{65\text{min}}{46.5\text{m}^2}A_w = 82.4$ min, for a total of 126.6 min. For simplicity, we round this cleaning time down to two hours (see §3.6), and assume that a 1-log reduction can be achieved on the surfaces in two hours, so that $k_f(t) = k_w(t) = \frac{\ln 10}{2\text{hr}} = 1.15/\text{hr}$ for $t \in [0, T)$.

3.4 Air Cleaning

The parameter $k_a(t)$, sometimes called the air exchange rate, is typically calculated by dividing the volumetric flow rate Q by the room volume V, and then multiplying this ratio by a mixing factor, which can range from about 0.1 to 0.5, depending upon the ventilation characteristics of the room [8]. We assume that an air exchange rate of $k_a(t) = 10/\text{hr}$, which is typical during an asbestos cleanup, is achieved for $t < T$.

3.5 Duct Modeling

To assess the source rate from the duct, we first need to estimate how many spores are initially deposited in the duct. Consider a straight duct of height and width $W = 0.4$ m [8], and length L, through which air is flowing horizontally at rate v_x. The duct efficiency, η, which is the fraction of spores entering the duct that are deposited there, is given by $1 - \exp\left(-\frac{v_y L}{v_x W}\right)$ under well-mixed conditions and by $\frac{v_y L}{v_x W}$ under laminar conditions [8]. If we assume that all spores entering the building do so through the ducts (many will enter through windows, doors and other gaps) then the number of spores deposited in the duct is $\frac{\eta}{1-\eta}$ times the number of spores in the room. We assume the horizontal duct velocity is $v_x = 1000$ ft/min=5.08 m/s, which is at the low end of values reported for industrial applications (Table 6.6 in [8]). With any reasonable value of L, the duct efficiencies under well-mixed and laminar conditions nearly coincide and are very small, and for concreteness we use the laminar efficiency, $\frac{v_y L}{v_x W}$. To be conservative, we set $L = 50$ m, which is considerably longer than most ducts, and obtain an efficiency of 1.95×10^{-3}.

However, many ducts are curved. Consider a curved duct of width $W = 0.4$ m, inner radius $r_1 = 0.3$ m and outer radius $r_2 = 0.7$ m [8]. Under the well-mixed, irrotational flow model [8], the efficiency of this curved duct (due solely to the curvature, ignoring gravitational settling) that traverses the angle θ is $1 - e^{-CKS\theta}$, where $C = 1.05$ is the Cunningham slip factor, $K = \frac{r_2 - r_1}{r_2[\ln(r_2/r_1)]^2} = 0.796$, and the average Stokes number $S = \frac{\rho_p D_p^2 v_x}{18 v_d r_2} = 5.61 \times 10^{-5}$. The amount of total curvature in ducts varies widely, and we assume 360 degrees in total (i.e., $\theta = 2\pi$), which has efficiency 2.95×10^{-4}. To be conservative, we add these two efficiencies (which overstates the efficiency, due to the possibility of double counting deposited particles) and set $\eta = 2.24 \times 10^{-3}$ and $\frac{\eta}{1-\eta} = 2.25 \times 10^{-3}$.

A room that has a deposition of D spores/m^2 in §1 has $0.027DA_w + 0.973DA_f$ spores in the room just after the attack. Hence, the number of spores deposited in the duct just

after the attack is

$$\tilde{D} = \frac{\eta}{1-\eta}[0.027DA_w + 0.973DA_f]. \tag{13}$$

We assume that these spores disengage from the duct and enter the room at a rate α per unit time. If remediation begins $\tau_d = 7$ days after the attack, then the room concentrations at the time cleaning begins are $c_a(0) = 0$,

$$c_w(0) = 0.027D + \frac{0.027\tilde{D}}{A_w}\int_0^{\tau_d} \alpha e^{-\alpha s}\, ds = 0.027D + \frac{0.027\tilde{D}(1-e^{-\alpha\tau_d})}{A_w}, \tag{14}$$

$$c_f(0) = 0.973D + \frac{0.973\tilde{D}(1-e^{-\alpha\tau_d})}{A_f}, \tag{15}$$

and the duct term in equation (1) is given by

$$d(t) = \frac{\tilde{D}\alpha e^{-\alpha(\tau_d+t)}}{V}. \tag{16}$$

The parameter α is largely unknown and depends upon the age and composition of the duct. Hence, to be conservative, we attempt (via the following simplified model) to choose the value of α that maximizes the number of anthrax cases. Let $x(t)$ denote the number of spores from the duct that are in the room at time t. Then at the time cleaning begins, we have $x(0) = \tilde{D}(1-e^{-\alpha\tau_d})$. For simplicity, we ignore the surface cleaning and assume that these spores die at rate $k_a(t)$, which is 10/hr for $t < T$ and 1.8/hr for $t \geq T$ (this is the average during the post-reoccupation period; see §3.7). Hence, the quantity $x(t)$ evolves according to

$$\dot{x}(t) = \tilde{D}\alpha e^{-\alpha(\tau_d+t)} - k_a(t)x(t). \tag{17}$$

Assuming a reoccupation period of 10 years, we analytically solve the linear ODE (17) and integrate its solution from time $T = 21$ days (which represents a typical value, given our goal of full reoccupation by 42 days) to 10 years, and then computationally maximize $\int_T^{10\text{ yr}} x(t)\, dt$ to get $\alpha = 1.34 \times 10^{-3}$/hr.

3.6 Sampling and Cleaning Strategies

Our strategy employs an initial pre-cleaning sample followed by successive rounds of cleaning and sampling, and contains two decision variables, one dictating how much samping to do and one specifying how clean the room should be. Each sampling includes n_s floor samples per room; n_s is a decision variable that allows us to assess the appropriate amount of sampling. A room's microenvironment will lead to unpredictable spatial heterogeneity of spore concentrations within the room. Rather than use a spatial model to capture this statistical uncertainty [19], we assume that samples are log-normally distributed with median e^{μ} equal to the true spore concentration on the floor, which is given by $c_f(0)$ in (15) if sampling occurs before cleaning is initiated and by $c_f(t)$ in (3) if sampling occurs at time $t > 0$. The dispersion is $e^{\sigma} = 10^{1/4}$ (i.e., the ln of the samples are normal with mean μ and standard deviation σ), so that 95% of the samples fall within one order of magnitude (i.e., between $1/\sqrt{10}$ of the median and $\sqrt{10}$ of the median). The samples from the Hart Senate Office Building appear to have somewhat more variability than this, although they were taken from an area larger than the size of our generic room. The initial pre-cleaning samples are denoted by $(Y_{01}, \ldots, Y_{0n_s})$, and our point estimate of $c_f(0)$ is

$$\hat{D}_0 = \exp\left(\frac{\ln(Y_{01} \cdots Y_{0n_s})}{n_s}\right). \tag{18}$$

We assume that vacuuming the room surfaces and contents takes $\tau_v = 2$ hr per room, and each worker cleans two rooms per day; as explained in the main text, six hours per ten-hour shift are required for rest, rehydration, and dealing with protective gear. Cleaning and testing are on the following 48-hour cycle. The initial testing takes place at time 0, the first cleaning takes place during the interval [24,26] hours and, if need be, every 48 hours thereafter. Additionally, any desired testing takes place at multiples of 48 hours (i.e., $t = 48, 96, \ldots$). The 24-hour delay between the initation of cleaning and subsequent testing (if need be) allows most of the spores to resettle after cleaning, while the 24-hour

delay between testing and subsequent cleaning (if need be) permits test results, which are typically known within about 18 hours, to be received before deciding whether subsequent cleaning is required. We implicitly assume that each cleaner works on two sets of two rooms on alternate days so as to avoid idleness while waiting for test results from the first set of rooms. Let $\tau_a = 24$ hr, which represents the time between a test and the next cleaning (if need be) and between a cleaning and the next test (if need be). Let n_r be the number of days until reoccupation, i.e., reoccupation occurs at time

$$T = n_r \tau_a. \tag{19}$$

Because reoccupation occurs after a final test result (see below), n_r must be an odd number. Hence, the number of vacuumings will be $(n_r - 1)/2$, and the indicator function for cleaning is given by

$$I_f(t) = I_w(t) = \begin{cases} 1 & \text{if} \quad t \in \Big\{[\tau_a, \tau_a + \tau_v), [3\tau_a, 3\tau_a + \tau_v), \ldots, [(n_r - 2)\tau_a, (n_r - 2)\tau_a + \tau_v)\Big\}; \\ 0 & \text{if} \quad t \in \Big\{[0, \tau_a), [\tau_a + \tau_v, 3\tau_a)), \ldots, [(n_r - 3)\tau_a + \tau_v, (n_r - 2)\tau_a)\Big\}. \end{cases} \tag{20}$$

To allow more highly contaminated rooms to receive more intensive cleaning, we let n_r vary according to the estimated deposition. In fact, n_r (and hence T) will be a random variable because of the statistical uncertainty in the measurement samples. More specifically, in each round of cleaning and sampling, we vacuum the room on alternate days (i.e., once every 48 hours) until it is believed that the floor concentration is below the threshold parameter $\bar{c}_f$, which is the second decision variable in our strategy. Then we take n_s samples in an attempt to confirm that the floor concentration is indeed below the threshold. If our new estimate is below the threshold, then vacuuming ceases. Otherwise, we use the new estimate to determine how many more vacuumings are needed to get below the threshold; in the latter case, we then perform these vacuumings and retest. This process of cleaning and sampling is repeated until a post-cleaning sample produces an estimate that is below the threshold. Hence, we assume that the decision maker has access to the compartmental model

in (1)-(3) and the current point estimate, but not the exact current state. This implicitly assumes that the managers have a reasonably good estimate of the number of air exchanges per hour ($k_a(t)$) and the vacuuming efficiencies ($k_f(t)$, $k_w(t)$), which is likely the case for an experienced asbestos cleanup crew, for example. To describe this process mathematically, we note that in round l, we perform $n_l/2$ vacuumings until our estimated floor concentration next drops below the threshold parameter $\bar{c}_f$; by definition, n_l is an even number. Then we take our l^{th} set of post-cleaning samples, $(Y_{l1}, \ldots, Y_{ln_s})$, which are log-normally distributed with median $e^{\mu} = c_f(\sum_{k=1}^{l} n_k\tau_a)$, thereby generating the estimated post-cleaning floor concentration of

$$\hat{D}_l = \exp\left(\frac{\ln(Y_{l1}\cdots Y_{ln_s})}{n_s}\right). \tag{21}$$

Let $c_a(t;\hat{D}_l)$, $c_w(t;\hat{D}_l)$ and $c_f(t;\hat{D}_l)$ be the estimated room concentrations at time $t \in [\sum_{k=1}^{l} n_k\tau_a, \sum_{k=1}^{l+1} n_k\tau_a)$, which is the time interval between the lth and $l+1^{\text{st}}$ post-cleaning samples. These quantities are computed as follows. The true state of the system at the time of the lth post-cleaning sample is $c_a(\sum_{k=1}^{l} n_k\tau_a)$, $c_w(\sum_{k=1}^{l} n_k\tau_a)$ and $c_f(\sum_{k=1}^{l} n_k\tau_a)$, as computed by (1)-(3). After taking the measurements leading to $\hat{D}_l$ in (21) at time $\sum_{k=1}^{l} n_k\tau_a$, the estimated floor concentration at time $\sum_{k=1}^{l} n_k\tau_a$ is by definition

$$c_f(\sum_{k=1}^{l} n_k\tau_a;\hat{D}_l) = \hat{D}_l. \tag{22}$$

We assume that air and wall concentrations at time $\sum_{k=1}^{l} n_k\tau_a$ are also misestimated by the factor $\frac{\hat{D}_l}{c_f(\sum_{k=1}^{l} n_k\tau_a)}$, which gives

$$c_a(\sum_{k=1}^{l} n_k\tau_a;\hat{D}_l) = \frac{\hat{D}_l}{c_f(\sum_{k=1}^{l} n_k\tau_a)} c_a(\sum_{k=1}^{l} n_k\tau_a), \tag{23}$$

$$c_w(\sum_{k=1}^{l} n_k\tau_a;\hat{D}_l) = \frac{\hat{D}_l}{c_f(\sum_{k=1}^{l} n_k\tau_a)} c_w(\sum_{k=1}^{l} n_k\tau_a). \tag{24}$$

The quantities $c_a(t;\hat{D}_l)$, $c_w(t;\hat{D}_l)$ and $c_f(t;\hat{D}_l)$ for $t \in [\sum_{k=1}^{l} n_k\tau_a, \sum_{k=1}^{l+1} n_k\tau_a)$ are computed by solving (1)-(3) starting at time $\sum_{k=1}^{l} n_k\tau_a$ with initial conditions given by the estimated concentrations in (22)-(24) rather than the true concentrations.

We can now define the number of days until reoccupation, n_r, which is a random variable given by

$$n_r = \begin{cases} 1 & \text{if} \quad \hat{D}_0 < \bar{c}_f; \\ 2(\sum_{l=1}^{j} n_l) + 1 & \text{if} \quad \begin{cases} \text{for } l = 1, ..., j \begin{cases} \hat{D}_{l-1} \geq \bar{c}_f; \\ c_f(i\tau_a; \hat{D}_{l-1}) \geq \bar{c}_f, i = 0, 2, 4..., n_l - 2; \\ c_f(n_l\tau_a; \hat{D}_{l-1}) < \bar{c}_f; \end{cases} \\ \hat{D}_j < \bar{c}_f. \end{cases} \end{cases} \tag{25}$$

3.7 Post-reoccupation Cleaning and Cumulative Dose

We assume that the contaminated zone is reoccupied at the density of $\gamma = 0.075$ people/ m^2 of floor space [2], which is one person per generic room. These reoccupants reside in these buildings for 12 hours per day, breathing at rate $b = 138$ m^3/hr [20] from a (sitting or sleeping) height of 1 m. To be conservative, we assume that these rooms experience active conditions during these 12 hours and experience semi-quiescent conditions during the other 12 hours. That is, we assume that for $t > T$ measured in hours,

$$f_w(t) = \begin{cases} \tilde{f}_w^a & \text{if} \quad t \in [T + 24n, T + 24n + 12), n = 0, 1, \ldots; \\ \tilde{f}_w^s & \text{if} \quad t \in [T + 24n + 12, T + 24(n + 1)), n = 0, 1, \ldots; \end{cases} \tag{26}$$

$$r_w(t) = \begin{cases} \tilde{r}_w^a & \text{if} \quad t \in [T + 24n, T + 24n + 12), n = 0, 1, \ldots; \\ \tilde{r}_w^s & \text{if} \quad t \in [T + 24n + 12, T + 24(n + 1)), n = 0, 1, \ldots; \end{cases} \tag{27}$$

$$r_f(t) = \begin{cases} \tilde{r}_f^a & \text{if} \quad t \in [T + 24n, T + 24n + 12), n = 0, 1, \ldots; \\ \tilde{r}_f^s & \text{if} \quad t \in [T + 24n + 12, T + 24(n + 1)), n = 0, 1, \ldots. \end{cases} \tag{28}$$

The deposition and reaerosolization parameter values during the semi-quiescent post-reoccupation periods are assumed to be identical to the semi-quiescent parameter values during the cleanup period, i.e., $\tilde{f}_w^s = f_w^s$, $\tilde{r}_w^s = r_w^s$ and $\tilde{r}_f^s = r_f^s$. However, the walls are not cleaned during the active post-reoccupation periods (see below), and are likely to experience much less reaerosolization than in the active cleaning period. On the other hand, spores are more apt to deposit on the walls during active conditions than semi-quiescent conditions. Hence, we

assume that $\frac{\tilde{r}_w^a}{\tilde{r}_f^a} = \frac{\tilde{r}_w^s}{r_f^a} = 8.79 \times 10^{-3}$, i.e., the ratio of wall-to-floor reaerosolization during the active reoccupation period is the same as the wall reaerosolization during the semi-quiescent cleanup period divided by the floor reaerosolization during the active cleanup period. We solve this equation simultaneously with (8)-(10) and obtain the values of $\tilde{r}_w^a$, $\tilde{r}_f^a$ and $\tilde{f}_w^a$ that appear in Table 1.

We assume that post-reoccupation cleaning (performed or paid by the reoccupants, without protective gear) occurs at lower levels than during the remediation period. A portable HEPA filter with a fan operated at a flow rate of 404 m^3/hr, which is representative of commercial air cleaners, achieved an air exchange rate of 3.0/hr in a room the size of our generic room [9]. We assume that the HEPA filters and fans achieve an air exchange rate of $\tilde{k}_a^a = 3.0$/hr during the 12 hours of active conditions (i.e., the fans are left running while people are present), and achieves an air exchange rate of $\tilde{k}_a^s = 0.5$/hr during the other 12 hours in a day. That is,

$$k_a(t) = \begin{cases} \tilde{k}_a^a & \text{if } \ t \in [T + 24n, T + 24n + 12), n = 0, 1, \ldots; \\ \tilde{k}_a^s & \text{if } \ t \in [T + 24n + 12, T + 24(n + 1)), n = 0, 1, \ldots. \end{cases} \tag{29}$$

We assume that the floor, which has area $A_f = 16.05$ m^2, is HEPA vacuumed (dry rather than wet) once per $\tilde{\tau}_a = 7$ days for $\tau_f = 10$ minutes. To derive the cleaning rate $k_f(t)$, we assume that the post-reoccupation vacuum is half as efficient as the wet vacuum used during the cleanup, so that $\frac{65\text{min}}{46.5\text{m}^2}$ achieves only a 35% reduction in spores. Therefore, for $t \geq T$, we have $k_f(t) = \frac{-\ln(0.65)46.5\text{m}^2}{A_f 65\text{min}} = 1.15$/hr, which coincidentally is the same as $k_f(t)$ during cleanup. Because $e^{-k_f(t)\tau_f} = 0.825$, each round of post-reoccupation vacuuming only removes 17.5% of the remaining spores. No vacuuming of the walls or ceilings occurs during the reoccupation period. That is, for $t > T$, we set $I_w(t) = 0$ in (2) and change $I_f(t)$ in (3) to

$$I_f(t) = \begin{cases} 1 & \text{if } \ t \in \Big\{[T + n\tilde{\tau}_a, T + n\tilde{\tau}_a + \tau_f), \ n = 1, 2, \ldots\Big\}; \\ 0 & \text{if } \ t \in \Big\{[T, T + \tilde{\tau}_a), [T + n\tilde{\tau}_a + \tau_f, T + (n + 1)\tilde{\tau}_a), \ n = 0, 1, \ldots\Big\}. \end{cases} \tag{30}$$

We calculate the number of spores inhaled by each reoccupant over a 10-year horizon by solving the ODE system (1)-(3) for $t > T$ and converting the average air concentration $c_a(t)$ into the air concentration at the height of 1 m by multipling $c_a(t)$ by the factor

$$\frac{ae^{-a}}{\frac{\int_0^H ae^{-aH}}{H}} = \frac{Hae^{-a}}{1-e^{-aH}} = 0.716. \tag{31}$$

Hence, if we let s denote the number of spores inhaled by a reoccupant over a post-reoccupation period of ten years, and define the indicator residential function

$$I_r(t) = \begin{cases} 1 & \text{if} \quad t \in [T + 24n, T + 24n + 12), n = 0, 1, \ldots; \\ 0 & \text{if} \quad t \in [T + 24n + 12, T + 24(n+1)), n = 0, 1, \ldots, \end{cases} \tag{32}$$

then

$$s = \frac{Hae^{-a}b}{1-e^{-aH}} \int_T^{10 \text{ yr}} c_a(t) I_r(t)\ dt. \tag{33}$$

Note that s is a random variable because the lower integration limit T and the air concentration $c_a(t)$ depend on the sampling results.

3.8 Vaccine Coverage, Efficacy and Cost

The current vaccine is only licensed for, and has only been tested on, people from 18 to 65 years of age [21]. The vaccine is contraindicated for people with prior hypersensitivity or other severe reaction to any anthrax vaccine or those who have recovered from a prior clinical exposure. Precautions would apply to immunocompromised patients and those on immunosuppresant therapy, and those with a history of hypersensitivity to other medication. In addition, people who are pregnant or breastfeeding, have an infection/febrile illness or are on a short course of steriods should delay taking the vaccine [22]. To be conservative, we assume that a fraction $f_v = 0.85$ of the reoccupants are vaccinated, leaving 15% of the population, including young children, people over 65, and the immunocompromised, unvaccinated. The Working Group on Civilian Biodefense suggests that the US vaccine is likely

to be safe and effective in children [21]; hence, it is more likely that all noncontraindicated people would be offered the vaccine, and that the vaccine would be effective for more than 15% of the population. For the 85% vaccinated population, we assume the vaccine is fully protective, and causes no inhalational anthrax cases for the reoccupants, regardless of the spore levels. Because it is not practical to keep people on prophylactic antibiotics indefinitely, we assume that the 15% unvaccinated reoccupants receive no medical protection. The vaccine, which requires a series of six shots over 18 months plus an annual booster [21], is assumed to cost $c_v = \$20$ per person.

3.9 The Dose-Response Model

We need a dose-response curve that maps the cumulative dose in (33) into a response. The most widely accepted model is a probit model with a slope of 0.7 probits per log dose and an ID_{50} of 8000 spores [23]; i.e., the probability that someone who inhales s spores becomes infected is $\Phi(0.3 \ln s - 2.7)$, where $\Phi(\cdot)$ is the standard normal cumulative distribution function. This probit slope is from Glassman's primate study [24] and the ID_{50} is an estimate from the US Department of Defense [25]. There is considerable uncertainty on the low end of the dose-reponse curve. Haas [26] considers three data sources: Glassman's unpublished data (1236 animals, lowest dose considered is about ID_{20}) [24], Druett's monkey study (72 animals, range from 70,000 to 400,000 spores) [27], and Brachman's study (120 monkeys, range from 1000 to 25,000 spores) [28]. He argues that an exponential model is a better fit to the latter two studies than the probit model, and also that the probit model overestimates the fraction infected. However, Glassman's study is probably the most reliable, since it uses a large sample size and controlled conditions. In addition, Dahlgren [29] claims that goat-hair mill workers routinely inhaled about 500 (sub 5 micron) anthrax spores per shift without getting infected. Hence, people may develop immunity if exposed at low levels for

long periods. More recently, the 94-year old CT woman who died from inhalation anthrax without any evidence of anthrax in her house suggests that an elderly person can get infected from several spores [30]. This case is more consistent with a slope of 0.7 than of 1.4: the probability of someone getting infected from 5 spores is 0.013 if the slope is 0.7 (note that hundreds of people probably received cross-contaminated letters in 2001), but is only 4×10^{-6} if the slope is 1.4.

Because the dose-response curve for our model is for those who are not vaccinated, the probit model discussed above may underestimate the fraction of cases from these subpopulations. Consequently, we assume that the 15% unvaccinated are sampled randomly from the bottom 30% of the probit dose-response curve described above, so that the probability $p(s)$ that an unvaccinated reoccupant is infected by inhaling s spores is

$$p(s) = \min\left\{\frac{\Phi(0.3 \ln s - 2.7)}{0.3}, 1\right\}. \tag{34}$$

3.10 Computation of Cases, Cost and Total Cleaning Time

From §1, we have $n(D)$ square meters of indoor space that have a deposition of D spores/m^2. For a deposition of D spores/m^2, equations (19), (25) and (33) give the random number of spores inhaled over a 10-year reoccupancy, equation (34) gives the dose-response curve for the 15% of reoccupants that are unvaccinated, and γ is the population density of reoccupants. Taken together, if we define $f(s; D)$ to be the probability density function of the number of inhaled spores s in (33) for a fixed value of D, then the expected number of inhalation anthrax cases is

$$(1 - f_v)\gamma \int \Big(\int p(s) f(s; D)\, ds \Big) n(D)\, dD, \tag{35}$$

where the inner integral represents the likelihood of infection given the dose D, and the outer integration is over the entire dose range in the exposed region. Because the function $f(s; D)$ does not have an explicit analytical form, we resort to Monte Carlo simulation to

compute (35). More specifically, we simulate (35) 50 times, which results in the 95% half-confidence interval for the number of anthrax cases to be less than 0.1 times the sample mean of the number of cases.

We assume that each Hazmat person is paid c_l = \$75/hr, which includes the use of the vacuums. According to §3.6, the labor cost to clean four generic rooms is $c_l(2\tau_v+\tau_p)(n_r-1)$, where $\tau_p = 6$ hr accounts for getting in and out of, and decontaminating, protective gear, and rest and rehydration. In addition, each environmental sample costs c_s =\$25, which includes a \$30/hr sampler obtaining 2.4 samples/hr (see the next paragraph), plus \$1 for shipping, plus \$11.50/sample for the laboratory cost. We assume that each portable HEPA cleaner costs c_h = \$250; everyone is assumed to already own a vaccum. Let us define $h(n_r;D)$ to be the probability density function of n_r as given in (25), and $g(j;D)$ to be the probability density function of the quantity j in (25) for fixed D, which is the total number of rounds of post-cleaning sampling. Then the total expected cost, which includes labor, sampling, HEPA cleaners and vaccines, to remediate the entire exposed region of A m^2 of floor surface area is

$$\frac{(2\tau_v+\tau_p)c_l}{4(13.38)}\int\Big(\int(n_r-1)h(n_r;D)\;dn_r\Big)n(D)\;dD+\frac{A}{13.38}c_h+f_v\gamma Ac_v$$
$$+\frac{c_sn_s}{13.38}\int\Big(\int(j+1)g(j;D)\;dj\Big)\;n(D)\;dD. \tag{36}$$

The Brentwood cleanup used about 300 Hazmat people (after attrition) and there are about 3000 licensed asbestos cleanup workers in New York State, many of whom could be recruited. We assume that $l_h = 1000$ Hazmat people are available to perform cleanup for $2\tau_v+\tau_p = 10$ hours per day. Hence, $4l_h$ rooms can be cleaned every n_r-1 days. In addition, $l_s = 200$ samplers require 10 min per sample over four hours plus six hours to rest, rehydrate, and put on and remove protective gear, leading to a throughput rate per sampler of $\mu_s = 24$ samples per day. We assume the bottleneck for the cleanup time can be either vacuuming or sampling. Hence, the expected number of days required to remediate the entire exposed

region is

$$\max\left\{\frac{1}{4(13.38)l_h}\int\Big(\int(n_r-1)h(n_r;D)\ dn_r\Big)n(D)\ dD,\ \frac{n_s}{\mu_s(13.38)l_s}\int\Big(\int jg(j;D)\ dj(D)\Big)n(D)\ dD\right\}. \tag{37}$$

References

[1] R. I. Sykes, S. F. Parker, D. S. Henn, C. P. Cerasoli, L. P. Santos, PC-SCIPUFF Version 1.1PD.1. Technical Documentation, ARAP Report 718, Titan Corporation, Princeton, NJ (1998).

[2] http://www.sanbornmap.com.

[3] V. P. Reshetin, J. L. Regens, Simulation modeling of anthrax spore dispersion in a bioterrorism incident. *Risk Analysis* **23**, 1135-1145 (2003).

[4] http://www.govexec.com/dailyfed/1103/11120cl.htm.

[5] United States Postal Service, Emergency preparedness plan for protecting postal employees and postal customers from exposure to biohazardous material and for ensuring mail security against bioterror attacks. (March 6, 2002).

[6] United States General Accounting Office, Captiol Hill anthrax incident: EPA's cleanup was successful; opportunities exist to enhance contract oversight. GAO-03-686 (June 2003).

[7] C. P. Weis, A. J. Intrepido, A. K. Miller, P. G. Cowin, M. A. Durno, J. S. Gebhardt, R. Bull, Secondary aerosolization of viable *bacillus anthracis* spores in a contaminated US Senate office. *JAMA* **288**, 2853-2858 (2002).

[8] R. J. Heinsohn, J. M. Cimbala, *Indoor Air Quality Engineering*, Marcel Dekker, Inc., New York (2003).

[9] Y. S. Cheng, J. C. Lu, T. R. Chen, Efficiency of a portable indoor air cleaner in removing pollens and fungal spores. *Aerosol Science and Tech.* **29**, 92-101 (1998).

[10] A. C. K. Lai, Particle deposition indoors: a review. *Indoor Air* **12**, 211-214 (2002).

[11] R. B. Gore, B. Durrell, S. Bishop, L. Curbishley, A. Woodcock, A. Custovic, High-efficiency particulate arrest-filter vacuum cleaners increase personal cat allergen exposure in homes with cats. *J. Allergy Clin. Immunol.* **111**, 784-787 (2003).

[12] J.-L. Sagripanti, A. Bonifacino, Comparative sporicidal effects of liquid chemical agents. *Applied Envir. Microbiology* **62**, 545-551 (1996).

[13] R. T. Toledo, F. E. Escher, J. C. Ayres, Sporicidal properties of hydrogen peroxide against food spoilage organisms. *Applied Microbiology* **26**, 592-597 (1973).

[14] G. Dychdala, Chlorine and chlorine compounds. In *Disinfection and sterilization and preservation*, S. S. Block, Ed., Lea and Febiger, Philadelphia, PA, 157-182 (1983).

[15] http://www.deconsolutions.com.

[16] http://www.envirofoam.com.

[17] T. Hamouda, M. M. Hayes, Z. Cao, R. Tonda, K. Johnson, C. Wright, J. Brisker, J. R. Baker, Jr., A novel surfactant nanoemulsion with broad-spectrum sporicidal activity against *Bacillus* species. *J. Infect. Dis.* **100**, 1939-1949 (1999).

[18] J. R. Kominsky, R. W. Freyberg, J. Chesson, W. C. Cain, T. J. Powers, R. C. Wilmoth, Evaluation of two cleaning methods for the removal of asbestos fibers from carpet. *Am. Ind. Hyg. Assoc. J.* **51**, 500-504 (1990).

[19] T. Schneider, O. Holm Peterson, A. Aasberg Nielson, K. Windfield, A geostatistical approach to indoor surface sampling strategies, *J. Aerosol Sci.* **21**, 555-567 (1990).

[20] W. S. Linn, C. E. Spier, J. D. Hackney, Activity patterns in ozone-exposed construction workers. (previously published in *J. Occup. Med. Toxicol.* **2**, 1-14 (1993)). *J. Clean Technol. Environ. Sci.* **3**, 101-114 (1993).

[21] T. V. Inglesby, T. O'Toole, D. A. Henderson, J. G. Bartlett, M. S. Ascher, E. Eitzen, M. Friedlander, J. Gerberding, J. Hauer, J. Hughes, J. McDade, M. T. Osterholm, G. Parker, T. M. Perl, P. K. Russell, K. Tonat, Anthrax as a biological weapon, 2002: updated recommendations for management. *JAMA* **287**, 2236-2252 (2002).

[22] Centers for Disease Control and Prevention. Use of anthrax vaccine in the United States. *MMWR* **49**, no. RR-15 (December 15, 2000).

[23] M. Meselson, J. Guillemin, M. Hugh-Jones, A. Langmuir, I. Popova, A. Shelokov, O. Yampolskaya, The Sverdlovsk anthrax outbreak of 1979. *Science* **266**, 1202-1208 (1994).

[24] H. N. Glassman, Discussion. *Bacteriol. Rev.* **30**, 657-659 (1966).

[25] Defense Intelligence Agency, *Soviet biological warfare threat*, DST-1610F-057-86 (U.S. Department of Defense, Washington, DC, 1986).

[26] C. N. Haas, On the risk of mortality to primates exposed to anthrax spores. *Risk Analysis* **22**, 189-193 (2002).

[27] H. A. Druett, D. W. Henderson, L. Packman, S. Peacock, Studies on respiratory infection. *Journal of Hygiene* **51**, 359-371 (1953).

[28] P. S. Brachman, Inhalation anthrax. *Ann. NY Acad. Sci.* **353**, 83-93 (1980).

[29] C. M. Dahlgren, L. M. Buchanan, H. M. Decker, S. W. Freed, C. R. Phillips, P. S. Brachman, *Bacillus anthracis* aerosols in goat hair processing mills. *Am. J. Hygiene* **72**, 24-31 (1960).

[30] K. S. Griffith, P. Mead, G. L. Armstrong, J. Painter, K. A. Kelley, A. R. Hoffmaster, D. Mayo, D. barden, R. Ridzon, U. Parashar, E. H. Teshale, J. Williams, S. Noviello, J. F. Per, E. E. Mast, D. L. Swerdlow, J. L. Hadler, Bioterrorism-related inhalational anthrax in an elderly woman, Connecticut, 2001. *EID* **9**, 681-688 (2003).

Parameter	Description	Value	Reference
A	Total exposed indoor floor area	5.73×10^7 m^2	§1
	Size of generic room	$12 \times 12 \times 8$ ft	§3.1
V	Room volume	32.62 m^3	§3.1
A_f	Floor surface area in room	16.05 m^2	§3.1
A_w	Walls surface area in room	58.86 m^2	§3.1
D_p	Spore diameter	3 μm	[21]
C	Cunningham slip factor	1.05	[8]
g	Acceleration of gravity	9.81 m/s^2	§3.2
ρ	Density of air	1.184 kg/m^3	§3.2
v_d	Dynamic velocity	1.83×10^{-5} kg m^{-1} s^{-1}	§3.2
ρ_p	Density of anthrax spores	283 kg/m^3	§3.2
	Inverse spore mass	2.5×10^{14} spores/kg	[21]
v_y	Gravimetric settling velocity	7.93×10^{-5} m/s	(4)
l_a	Surface adsorption parameter	1.82×10^{-4}/s	§3.2
$f_w^a, \tilde{f}_w^a$	Fraction deposited on walls (active)	$0.098, 9.55 \times 10^{-4}$	(8)-(11)
$f_w^s, \tilde{f}_w^s$	Fraction deposited on walls (semi-quiescent)	8.63×10^{-4}	(8)-(10),(12)
$r_w^a, \tilde{r}_w^a$	Reaerosolization from walls (active)	1.252/hr, 0.012/hr	(8)-(11)
$r_w^s, \tilde{r}_w^s$	Reaerosolization from walls (semi-quiescent)	0.011/hr	(8)-(10),(12)
$r_f^a, \tilde{r}_f^a$	Reaerosolization from floor (active)	1.252/hr, 1.387/hr	(8)-(11)
$r_f^s, \tilde{r}_f^s$	Reaerosolization from floor (semi-quiescent)	0.011/hr	(8)-(10),(12)
a	Exponential settling parameter	1.83/m	§3.2
f_a^a	Fraction reaerosolized (active)	0.72	§3.2
f_a^s	Fraction reaerosolized (semi-quiescent)	0.02	§3.2
W	Duct width	0.4 m	[8]
L	Duct length	50 m	§3.5
v_x	Duct air flow rate	5.08 m/s	[8]
r_1	Inner radius of curved duct	0.3 m	[8]
r_2	Outer radius of curved duct	0.7 m	[8]
S	Average Stokes number of spores	5.61×10^{-5}	§3.5
θ	Total curvature in ducts	2π	§3.5
η	Duct efficiency	2.24×10^{-3}	§3.5
α	Spore disengagement rate from duct	1.34×10^{-3}/hr	(17)
γ	Population density	0.075 people/m^2	3.7
b	Breathing rate	1.38 m^3/hr	[20]
	Breathing height	1 m	§3.7
	Dose-response model		(34)

Table 1: Values for non-remediation parameters.

Parameter	Description	Value	Reference
c_c	Cost to fumigate first square meter	\$609	§2
τ_c	Time to fumigate first square meter	0.082 hr	§2
	Chlorine dioxide learning rate	90%	§2
τ_d	Remediation delay	7 days	§3.1
$k_a(t),\ t < T$	HEPA air exchange rate during cleanup	10/hr	§3.7
e^{σ}	Dispersion of random floor samples	$10^{1/4}$	§3.6
τ_v	Vacuuming time per room	2 hr	§3.6
$k_w(t),\ t < T$	Wall cleaning rate during cleanup	1.15/hr	§3.6
$k_f(t),\ t < T$	Floor cleaning rate during cleanup	1.15/hr	[18], §3.6
n_s	Number of floor samples	Decision	§3.6
$\bar{c}_f$	Floor threshold level for vacuuming	Decision	§3.6
τ_a	Time interval between vacuuming and testing	24 hr	§3.6
$\tilde{k}_a^a$	Post-reoccupation air exchange rate (active)	3/hr	§3.7
$\tilde{k}_a^s$	Post-reoccupation air exchange rate (semi-quiescent)	0.5/hr	§3.7
$\tilde{\tau}_a$	Post-reoccupation time interval between vacuumings	7 days	§3.7
$k_f(t),\ t \geq T$	Post-reoccupation floor cleaning rate	1.15/hr	§3.6
τ_f	Post-reoccupation floor vacuuming time	10 min	§3.7
f_v	Fraction vaccinated	0.85	§3.8
c_v	Vaccination cost	\$20/person	§3.8
c_l	Hazmat salary	\$75/hr	§3.10
τ_p	Time for protective gear	6 hr	§3.10
c_h	Cost of portable HEPA air cleaner	\$250	§3.10
l_h	Number of Hazmat personnel	1000	§3.10
c_s	Sampling cost	\$25	§3.10
μ_s	Sampling rate per sampler	48/day	§3.10
l_s	Number of human samplers	200	§3.10

Table 2: Values for remediation parameters.
